# Chicago Medical Society

**Published:** 1888-09

**Authors:** 


REGULAR MEETING, June 4, 1888.

Dr. Albert B. Strong presented a paper in which he reported "Six cases of Old Dislocation of the Head of the Humerus."

In the first case the violence of the attempts at reduction caused a laceration of the integument in the axilla, from which there was a copious but easily controlled hæmorrhage. The patient died from sepsis thirty-six hours after the operation, and the autopsy revealed a partial rupture of both axillary artery and vein.

The second and third cases presented nothing of special interest. The fourth case was that of a woman thirty-five years old, and the dislocation occurred six weeks prior to the operation. Just as the surgeons had concluded to abandon the case as irreducible, reduction occurred as the arm was despairingly lowered to the side. Recovery.

The remaining cases presented no points of special interest.

In the course of the discussion of the paper, Dr. Clark Gapen reviewed the medico-legal aspect of such cases, and strongly indorsed Dr. Strong's emphasis of the importance of taking notes of such cases at the time of their occurrence. The law requires, first, ordinary skill; second, ordinary care and diligence; third, best judgment. Ordinary skill has been defined as that degree of knowledge and skill attained by the profession up to a reasonable time of the occurrence in question.

ABNORMAL FŒTUS.

Dr. J. H. Stowell presented a specimen of a child born at full term. The mother is a woman of 35 years of age, and the father 40; they have both been married previously; the father by his first marriage had seven children, all healthy and well-formed; the mother by her first marriage had five children, healthy and well-formed. "This child which I present to-night is the first fruit of the second marriage. The child is a male, born at full term, and has no lower limbs whatever; one arm is off at the elbow and has a thumb attached to side of the humerus; the other arm is full length, but with only three fingers, two of these joined together. The child was born alive but lived only a few hours. The parents were colored." They cannot account for this in any way. Nothing of special interest was elicited by the discussion.

The President reported two cases of vaginal hysterectomy. In the course of the report he said: "The first case is one of great interest. The patient was placed in the extreme lithotomy position, a vaginal retractor put in over the perineum and drawn down; large lock vulcellum forceps were placed on the cervix and the latter was drawn down as far as possible. Dividing the vagina from the cervix was an easy matter with the scissors, and then separating the tissues all around from the cervix with the finger nail was easily accomplished. Large snap-forceps were placed on the left broad ligament. I found I had not sufficiently freed the uterus from the rectum, and cut a little with the scissors, and did a thing I will never do again. I reduced the size of the right broad ligament sufficiently to be grasped with another forceps. The uterus was removed in eleven minutes, but I spent three quarters of an hour trying to arrest hæmorrhage. It seemed as though every shred of divided tissue was bleeding. After my first case of hysterectomy I had constructed a very large retractor, whose blade is five and a half inches long by two and a quarter inches wide, with a suitable handle placed at right angles. The retractor was introduced into the vagina, flat surface downwards, and the rectum's anterior wall was perfectly exposed, and the bleeding artery was quickly exposed and seized. I had cut it off in freeing the rectal attachment to the uterus with the scissors. Forceps were placed upon this vessel, which completed the arrest of the hæmorrhage. In addition to the two large lock forceps I had thirteen others in the wound, which were taken off in 24 hours. The larger ones were taken off in 48 hours. The patient had no bad symptoms, and left the hospital in three weeks and two days.

The second case was a woman 42 years of age, and had borne several children. The removal of the uterus was determined upon, because of a rapidly growing carcinoma. This organ was small in size, and when exposed and drawn down with the vulcellum forceps it was brought easily to the mouth of the vagina. The vaginal wall was separated and the uterus brought down and removed in eight minutes. Four forceps only were left in this case, the hæmorrhage being easily checked. The patient was taken out of the operating room, completely dressed and ready for bed in 32 minutes after the commencement of the operation. She has gotten along without a bad symptom. The forceps were taken off in 48 hours. There was no rise of temperature except in the afternoon, when the temperature would go up to about 100, and go back again in the morning, showing that that there was no blood-poisoning going on. There was one peculiarity in this case. The operation was done at 10.30 o'clock Sunday morning; orders were left to catheterize the patient every six hours. She was catheterized shortly after the operation, but no urine was drawn, and again at six o'clock, without result. Just before completing these operations it is my custom to inject a solution of milk and water into the bladder, for the purpose of detecting any rupture of the bladder, and it revealed that the bladder was not ruptured; I knew, also, that I had not included the ureters in the forceps, because I kept too far away from them. Therefore, I felt that it must be suppression of urine, the trouble being in the kidneys. I saw the patient that night between nine and ten o'clock, and the nurse said the only thing she complained of was pain in the back, referred to the renal region. Poultices were ordered, and the next morning at six she was catheterized and a drachm and a half of urine only was obtained. A hypodermic injection of a half grain of pilocarpine was then administered, and I catheterized her four and a half hours later and drew off fourteen ounces of urine. With the exception of that one apparent bad symptom, the patient has had an uninterrupted recovery.

Professor J. A. Robison reported the following cases:

ANNUALLY RECURRING URTICURIA OF SEVERAL MONTHS' DURATION.

Miss G., æt. 29, has always been in good health until June, 1886. Menses appeared at 16 years of age, always been regular. Bowels generally regular, appetite small but variable. Has always been well nourished.

In June, 1886, an eruption resembling urticuria appeared on the arms, limbs, and body. It commenced with intense burning and itching, worse at night, intensified by heat, and causing loss of sleep. As a rule, the eruption would appear in the afternoon and disappear about 4 a. m. The result of this was the patient only slept from 4 a. m. till 9 a. m. each day. The eruption recurred daily from June, 1886, until February, 1887, when it disappeared after a two weeks' course of treatment. She was free from all trouble until May 15, 1887, when it reappeared and lasted until December 10.

May 13, 1788, she again visited him with the eruption out in full battle array. It appeared the night before at 7 p. m. and disappeared at 5 a. m.

BROMIDE ERUPTION OCCURRING IN AN INFANT.

This was a clearly defined case, but possessed no special interest beyond the fact of its existence and the unquestionable character of the diagnosis.

